# Supplementary material for: Two-Dimensional Gallium Sulfide Nanoflakes for UV-Selective Photoelectrochemical-type Photodetectors
Source: J Phys Chem C Nanomater Interfaces. 2021 May 26;125(22):11857–66. doi: 10.1021/acs.jpcc.1c03597 (PMC8279705; doi:10.1021/acs.jpcc.1c03597)
Supplement: Supplementary file 1 — jp1c03597_si_001.pdf [file jp1c03597_si_001.pdf]

Supporting Information

# Two-dimensional Gallium Sulfide Nanoflakes for UV-selective Photoelectrochemical-type Photodetectors

*Marilena I. Zappia,* [‡,§,Ɪ] *Gabriele Bianca,* [‡,†,⁑] *Sebastiano Bellani,*[*,§] *Nicola Curreli,*[#] *Zdeněk Sofer,*[//] *Michele Serri,*[†] *Leyla Najafi,*[§] *Marco Piccinni,*[†,⁑] *Reinier Oropesa-Nuñez,*[§,‡] *Petr Marvan,*[//] *Vittorio Pellegrini,*[§,‡] *Ilka Kriegel,*[#] *Mirko Prato,*[°] *Anna Cupolillo,*[Ɪ] *and Francesco Bonaccorso*[*,§,‡]

[§]BeDimensional Spa., via Lungotorrente Secca 3D, 16163 Genova, Italy

[†]Graphene Labs, Istituto Italiano di Tecnologia, via Morego 30, 16163, Genova, Italy

[Ɪ]Department of Physics, University of Calabria, Via P. Bucci cubo 31/C 87036, Rende (CS), Italy

[⁑]Dipartimento di Chimica e Chimica Industriale, Università degli Studi di Genova, via Dodecaneso 31, 16146 Genoa, Italy

[#]Functional Nanosystems, Istituto Italiano di Tecnologia (IIT), via Morego 30, 16163 Genova, Italy

[||]Department of Inorganic Chemistry, University of Chemistry and Technology Prague, Technická 5, 166 28 Prague 6, Czech Republic

[‡]Department of Material Science and Engineering, Uppsala University, Box 534, 75121 Uppsala, Sweden

[°]Materials Characterization Facility, Istituto Italiano di Tecnologia, via Morego 30, Genova 16163, Italy

## 1. Methods

### Materials

Gallium (Ga) (99.9999 %) and sulfur (S) (99.999 %) were purchased from Strem Chemicals Inc. USA. Sulfuric acid (99.999%), KOH (≥ 85% purity, ACS reagent, pellets), $Na_2SO_4$ (ACS reagent, ≥ 99.0%) were purchased from Sigma Aldrich.

### Synthesis and exfoliation of GaS crystals

Gallium sulfide (GaS) crystals were produced through direct synthesis from powders of Ga and S elements.[1] More in detail, an amount of Ga granules and S powder (15 g) with an elemental stoichiometry of 1:1 were loaded in a quartz glass ampoule (25 mm × 200 mm), which was subsequently evacuated (pressure <5 × $10^{-3}$ Pa) using a diffusion pump. The evacuated ampoule was then sealed using an oxygen-hydrogen torch. The ampoule was first heated in several steps below the melting point to react Ga and S and reduce generated pressure inside an ampoule (500°C / 48 hours, 600 °C / 48 hours and 800°C / 48 hours) and then heated up to 1000 °C (above the melting point of GaS: 965°C) for 1 h (heating rate = 5 °C $min^{-1}$). The samples were then cooled down to room temperature (cooling rate = 0.3 °C $min^{-1}$), producing the GaS crystal. The GaS flakes were produced by liquid-phase exfoliation (LPE)[2,3,4,] in anhydrous 2-propanol (IPA) of the pulverized GaS crystals, followed by sedimentation-based separation (SBS)[3,5,6] to remove unexfoliated material. Experimentally, 50 mg of bulk crystals were added to 50 mL of anhydrous IPA and ultrasonicated in a bath sonicator (Branson® 5800 cleaner, Branson Ultrasonics) for 15 h. The resulting dispersions were ultracentrifuged at 900 g (Optima™ XE-90 with a SW32Ti rotor, Beckman Coulter) for 20 min at 15 °C in order to separate un-exfoliated bulk crystals (collected as sediment) from the exfoliated

materials that remained in the supernatant. Then, 80% of the supernatant was collected by pipetting, getting an exfoliated material dispersion.

**Materials characterization**

Scanning electron microscopy (SEM) analysis of the as-synthetized crystal was performed using a Tescan Lyra microscope at 15kV and 0.5 nA. The samples were imaged without any metal coating or pre-treatment. The energy-dispersive X-ray spectroscopy (EDS) spectra were acquired with a microscope combined with an X-Max detector and INCA® system (Oxford Instruments), operating at 15kV and 0.8 nA. The samples were imaged without any metal coating or pre-treatment.

X-ray diffraction (XRD) measurements were acquired with a PANalytical Empyrean using Cu $K_\alpha$ radiation. The samples for XRD were prepared by depositing powder of GaS crystal onto $Si/SiO_2$ substrates.

Bright-field transmission electron microscopy (BF-TEM) images were acquired with a JEM 1400Plus (JEOL) TEM (thermionic source $LaB_6$ crystal), operating at 120 kV. The morphological and statistical analysis was performed by using ImageJ software (NIH) and OriginPro 9.1 software (OriginLab), respectively. The samples for the TEM measurements were prepared by drop-casting the as-prepared exfoliated material dispersions onto ultrathin C-on-holey C-coated Cu grids and rinsed with deionized water and subsequently dried overnight under vacuum. High resolution transmission electron microscopy (HR-TEM) measurements were carried out with an EFTEM Jeol 2200 FS microscope (Jeol, Japan), using a 200 keV acceleration voltage. Elemental maps and EDS spectra were acquired with SDD detector X-MaxN 80 TS from Oxford Instruments (England). The sample was prepared by drop casting the exfoliated dispersion on lacey C Supported Cu grids (200 mesh). The samples were dried at 50°C for 1 h before the measurements.

Atomic force microscopy (AFM) images were acquired with a XE-100 AFM (Park System, Korea) by means of PPP-NCHR cantilevers (Nanosensors, Switzerland) having a tip diameter inferior to 10 nm. The images were collected in intermittent contact (tapping) mode on an area of 5×5 $\mu m^2$ (1024×1024 data points) using a drive frequency of ~330 kHz and keeping the working setpoint above 70% of the free oscillation amplitude. The scan rate for the acquisition of the images was 0.2 Hz. Gwyddion 2.53 software (http://gwyddion.net/) was used for processing the images and the height profiles, while the data were analysed by using OriginPro 9.1 software. The latter was also used to carry out the statistical analysis on multiple AFM images (30 flakes) for all the tested samples. The samples were prepared by drop-casting the as-prepared GaS flake dispersion onto mica sheets (G250-1, Agar Scientific Ltd.) in $N_2$ and heating to 100°C for 15 min to dry the sample and remove adsorbates.

Raman spectroscopy measurements were performed by using a Renishaw microRaman Invia 1000 mounting a 50× objective, with an excitation wavelength of 633 nm and an incident power of 1 mW. For each sample, 50 spectra were collected. The samples were prepared by drop-casting the as-prepared GaS nanoflake dispersion onto Au-coated $Si/SiO_2$ substrates and subsequently dried under vacuum.

Diffuse reflectance spectroscopy (DRS) measurements were carried out on GaS flake dispersions (diluted or concentrated at various concentrations of GaS nanoflakes) using a Cary Varian 5000 UV/visible spectrometer with integrating sphere.

X-ray photoelectron spectroscopy (XPS) analysis was accomplished with a Kratos Axis UltraDLD spectrometer at a vacuum $< 10^{-8}$ mbar using a monochromatic Al Kα source operating at 20 mA and 15 kV and collecting photoelectrons from a 300 × 700 $\mu m^2$ sample area. The charge compensation device was not used. Wide spectra were acquired at pass energy of 160 eV and energy step of 1 eV, while high-resolution spectra were acquired at pass energy of 10 eV and energy step of 0.1 eV. The

samples were prepared by drop-casting the dispersion of GaS flakes on an Au-coated Si substrate in $N_2$ atmosphere while heating the substrate to 120°C. As-synthetized GaS crystals were stacked onto conductive carbon tape and cleaved prior analysis. The samples were then transferred from air to the XPS chamber. Data analysis is carried out with CasaXPS software (version 2.3.19PR1.0). The energy scale was calibrated by setting the lowest binding energy (B.E.) C1s component to 284.8 eV.

**Fabrication of the photoelectrochemical (PEC)-type and solid-state photodetectors**

For the PEC-type photodetectors, the photoelectrodes were produced by spray-coating the GaS flake dispersion onto graphite paper (PGS, Panasonic) mounted on a hot plate heated at 60 °C. The material mass loading was ~0.1 mg $cm^{-2}$. The photoelectrode area was 1.5×1 $cm^2$. The photoelectrodes were dried overnight at room temperature before the characterization.

For the solid-state photodetectors, a set of nine interdigitated source (S)/drain (D) electrodes were patterned on a highly p-doped Si chip covered with a 100 nm layer of thermal oxide. Each source/drain electrode pair consisted of 65 pairs of interdigitated fingers with an overlap of 40 μm and a gap L = 1 μm, which resulted in an overall channel width W = 5160 μm and an active area of W × L = 5160 $\mu m^2$ for photodetection. The electrode contacts were patterned by electron beam lithography (EBL) with poly(methyl methacrylate) (PMMA) resist, metal e-beam evaporation (5 nm Ti/30 nm Au) using magnets to not expose the PMMA, followed by lift-off. The device was completed by spray coating 10 mL of the as-produced GaS flake dispersion onto the entire surface of the chip while heating the substrate to 60 °C in air. The coated sample was placed under vacuum overnight at room temperature and then annealed at 200 °C for 30 min in Ar.

**Characterization of the photodetectors**

Scanning electron microscopy (SEM) analysis of the thin film deposited on a carbon paper electrode, was performed using a Raith150 Two system at 10kV and 0.2 nA. The samples were imaged without any metal coating or pre-treatment.

The electrochemical measurements on the PEC-type photodetectors were performed at room temperature in a flat-bottom fused silica cell using the three-electrode configuration of the potentiostat/galvanostat station (VMP3, Biologic), controlled *via* own software. A glassy carbon rod and a KCl-saturated Ag/AgCl were used as the counter-electrode and the reference electrode, respectively. The measurements were carried out in 200 mL of three different electrolytes, *i.e.*, 0.5 M $H_2SO_4$, 1 M $Na_2SO_4$, 1 M KOH. The pH of the electrolytic solutions was measured with Oakton ION 700 ISE/pH Meter. Before starting the measurements, the oxygen was purged from the electrolyte by flowing $N_2$ gas throughout the liquid volume using a porous frit. A constant, slight nitrogen flow is maintained afterward for the whole duration of the experiments. The Nernst equation: $E_{RHE} = E_{Ag/AgCl} + 0.059\times pH + E^0_{Ag/AgCl}$, where $E_{RHE}$ is the converted potential *vs.* RHE, $E_{Ag/AgCl}$ is the experimental potential measured against the Ag/AgCl reference electrode, and $E^0_{Ag/AgCl}$ is the standard potential of Ag/AgCl at 25 °C (0.1976 V *vs.* RHE), was used to convert the potential difference between the working electrode and the Ag/AgCl reference electrode to the reversible hydrogen electrode (RHE) scale. The LSV curves were acquired at 5 mV $s^{-1}$ scan rate, in both anodic and cathodic directions. The light-emitting diodes (LEDs) M275L4 (Thorlabs), M455L3 (Thorlabs), M505L3 (Thorlabs) and M625L3 (Thorlabs) were used as monochromatic source for wavelengths of 275, 455, 505, and 625 nm, respectively. The light intensity of the LEDs was adjusted through source meter (2612B Dual-Channel System SourceMeter, Keithley)-controlled LED driver (LEDD1B, Thorlabs). The illumination intensity of the LED was calibrated by using an optical power and energy meter (PM100D, Thorlabs). The responsivity of the photoelectrochemical (PEC)-type photodetectors was calculated by the ratio between the photocurrent density and the light intensity at fixed wavelength. The stability of the GaS photodetectors was evaluated by recording subsequent 20 LSV scans and measuring the corresponding responsivity.

The opto-electrical characterization of the solid-state photodetectors was performed at room temperature in air, using a shielded probe station equipped with a Keithley 2612 source meter. The measurement procedure was controlled via home-made software written in LabVIEW (National Instruments). The same LED sources used for the PEC-type photodetectors characterization were used to determine the photoresponse of the devices. The LED light was focused on the device with a collimating lens. The LED output power was controlled by the power supply (LEDD1B Thorlabs). Before measuring the device, the irradiance at the center of the focused light spot was determined with the following procedure: the light was focused on a pinhole (⌀ = 3.37 mm) and the power of the transmitted light was measured with a silicon photodiode (S120VC, Thorlabs) connected to a power meter (PM100D, Thorlabs), allowing the calculation of the irradiance at the center of the spot by dividing the power by the area of the pinhole.

## 2. Scanning electron microscopy-coupled energy-dispersive X-ray spectroscopy analysis of as-synthesized GaS crystals

**Table S1** shows the chemical composition of the as-synthesized GaS crystals, as estimated from the scanning electron microscopy (SEM)-coupled energy-dispersive X-ray spectroscopy (EDS) analysis reported in the main text (**Figure 1b,c**). As discussed in the main text, the SEM-coupled EDS analysis reveals a nearly ideal stoichiometry of the GaS crystals (Ga-to-S atomic ratio ~1.05),

**Table S1.** Elemental composition of the as-synthesized GaS crystals obtained from SEM-coupled EDS analysis.

| **Element** | **Atomic content (%)** |
|---|---|
| Ga | 51.3 |
| S | 48.7 |

## 3. Supplementary atomic force microscopy analysis

**Figure S1** shows an AFM image of representative LPE-produced GaS flakes, together with their height profiles. The thickness of the flakes ranges between 1.9 and 4.3 nm, indicating that there are few-layer flakes.

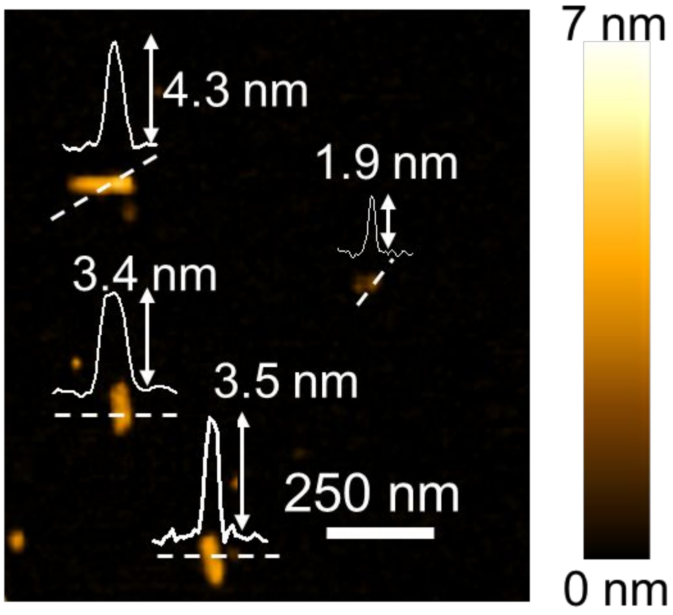


**Figure S1.** AFM image of representative GaS flakes. The height profiles of few-layer flake sections are also shown

## 4. Raman spectroscopy analysis of the bulk and exfoliated GaS crystals

**Figure S2** shows the quantitative statistical Raman analysis of the $A_{1g}^{1}$ peak position (calculated on 50 different spectra) for both bulk and exfoliated GaS crystals. As discussed in the main text, the peak position of $A_{1g}^{1}$ is a trusty indicator for simple and fast determination of the thickness of the GaS flakes,[7,8] since it is softened (red-shifted) while decreasing the number of the layers due to the reduced impact of the interlayer interaction on phonon restoring forces,[7,8] Therefore, the statistical results indicate that the $A_{1g}^{1}$ peak position for the GaS flakes is slightly red-shifted compared to the bulk case, indicating the successful exfoliation of the crystal through LPE method.

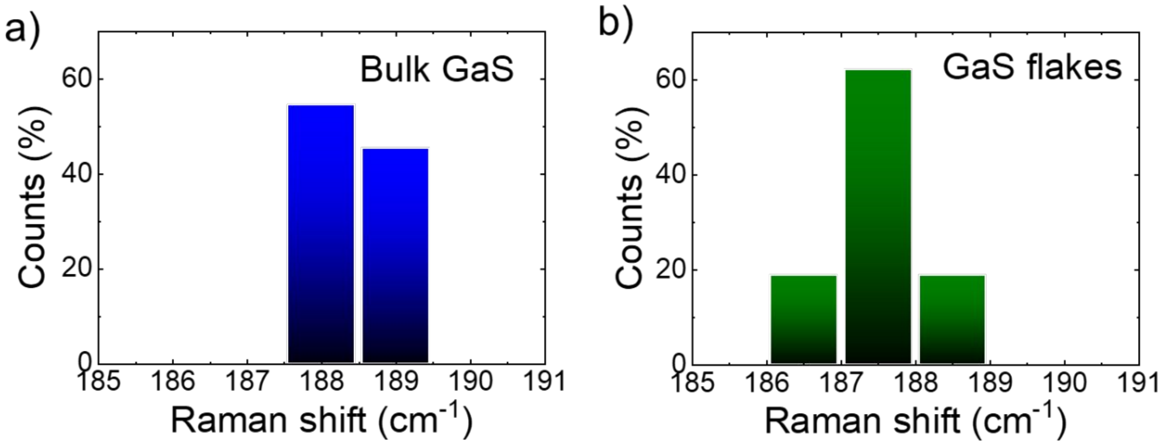


**Figure S2.** Statistical Raman analysis (calculated on 40 different spectra) of the $A_{1g}^{1}$ peak position for both a) bulk GaS and b) exfoliated GaS flakes

## 5. X-ray photoelectron spectroscopy analysis of the bulk and exfoliated GaS crystals

The composition and the chemical state of the bulk and exfoliated GaS crystals were evaluated by XPS measurements (**Figures S3** and **Figure S4**). The Ga 3d region in the bulk crystal exhibits a non-resolved doublet with fitted components at 20.7 and 21.2 eV, assigned to the GaS compound. Minor components at 19.7 eV and 21.7 eV can be assigned to small quantities of metallic Ga ($Ga^0$) and Ga oxides ($Ga\text{-}O_x$). The S 2p region reveals the well-resolved sulfide doublet with components at 163.2 and 164.4 eV, partially overlapping with the Ga 3s peak (161.5 eV).

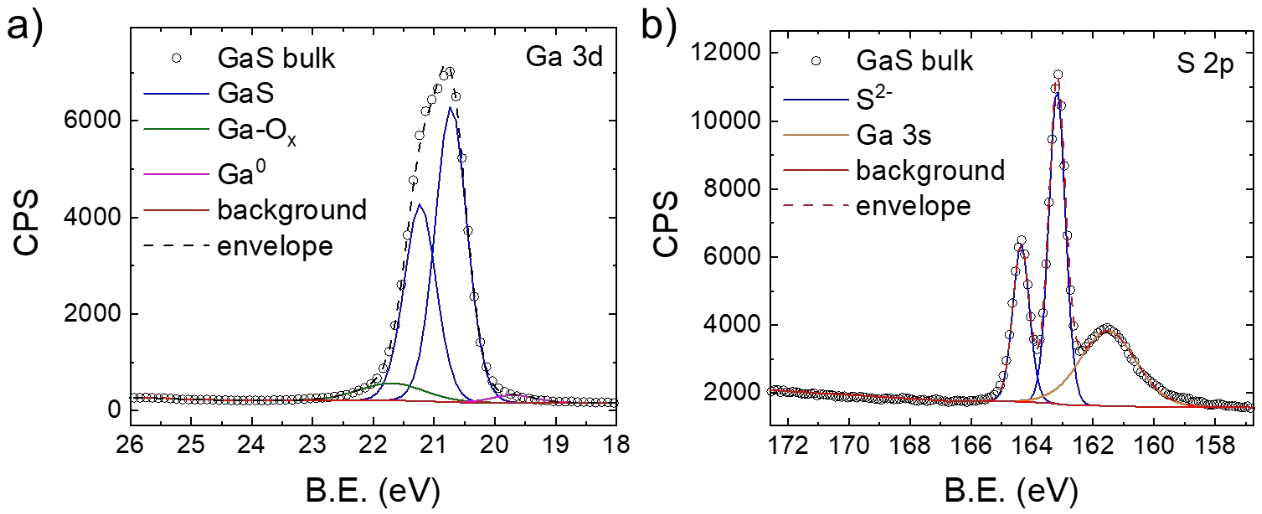


**Figure S3.** X-ray photoelectron spectroscopy analysis of the GaS bulk. a) Ga 3*d* region with the deconvoluted components. b) S 2p region with the deconvoluted components.

The spectra of the exfoliated GaS crystals are visibly broader compared to those of the bulk material. The Ga 3d region exhibits an increased occurrence of Ga oxides (B.E.~21.8 and 22.3 eV) on the surface of the crystals (~30% of surface Ga), which can be explained by the exposure of the sample to air prior to the measurement. The S 2p region also shows an additional component compared to the bulk, which could be assigned again to partial oxidation of the sample after exposure to air. This low-intensity component (~2% of the total S content) of oxidized sulfur species is observed as indicated by the peak at ~169 eV.

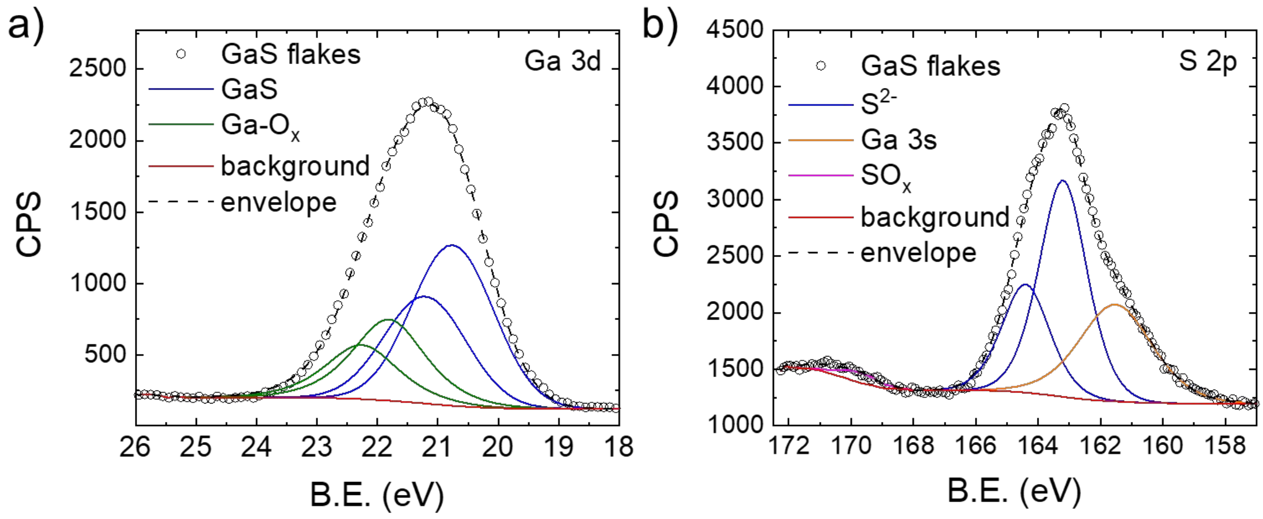


**Figure S4.** X-ray photoelectron spectroscopy analysis of the GaS nanoflakes. a) Ga 3*d* region with the deconvoluted components. b) S *2p* region with the deconvoluted components.

## 6. Characterization of solid-state photodetectors based on solution-processed GaS flake films

**Figure S5a** reports the sketch of the solid-state photodetector based on a GaS flake film deposited by spray coating onto Au interdigitated electrodes. **Figure S5b** shows the photocurrent as a function of the illumination intensity on the device at a wavelength of 275 nm using a source-drain voltage of 3 V. The dependence of the photocurrent on the illumination intensity is sublinear. Consequently, the responsivity decreases as the power increases, showing a value of 0.38 mA $W^{-1}$ at the illumination intensity of 1.3 mW $cm^{-2}$ (**Figure S5c**). The power-law dependence can be related to the dynamics of the traps and recombination centers determining the photoconductivity properties in nanostructured materials. A nonlinear dependence of the photocurrent on the light intensity can arise from the distribution of intra-bandgap traps and recombination centers[9,10] and the saturation of these states under strong light excitation.[9,11,12]

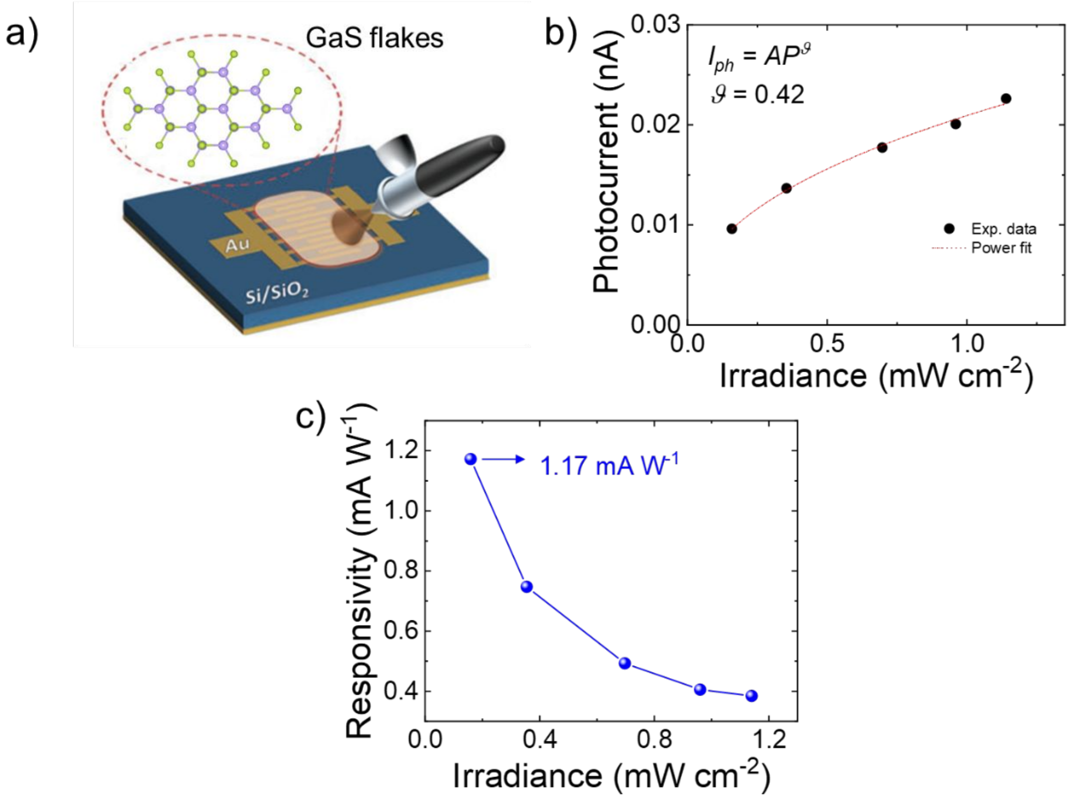


**Figure S5.** a) Sketch of the solid-state photodetectors based on spray-coated GaS flake film. b) Dependence of the photocurrent of the GaS photodetectors on the illumination intensity at the wavelength of 275 nm. The power-law fit of the experimental data is also shown. c) Dependence of the responsivity on the illumination intensity on the illumination intensity at the wavelength of 275 nm.

## 7. Supplementary scanning electron microscopy characterization of GaS photoelectrodes

**Figure S6** reports a top-view SEM image of a GaS photoelectrode, acquired with a lower magnification of the SEM image shown in **Figure 4c** of the main text. The GaS flake film shows a porous morphology.

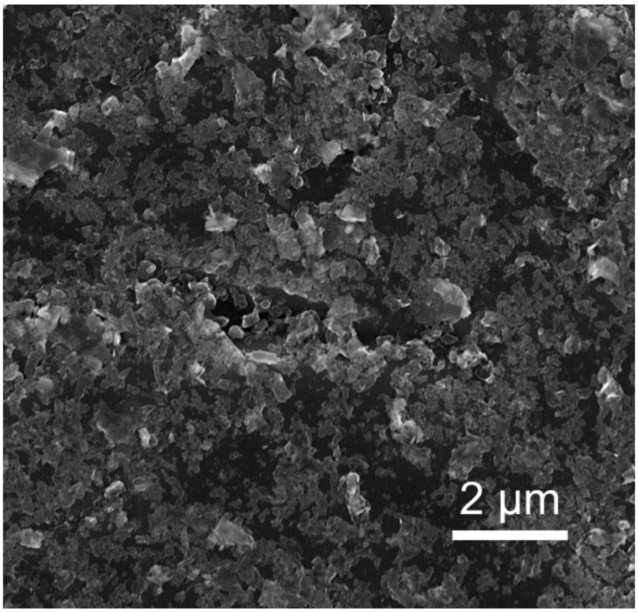


**Figure S6.** Top-view SEM image of a GaS photoelectrode.

## 8. Comparison between the responsivities of our PEC-type GaS photodetectors and other UV photodetectors based on solution-processed 2D materials.

**Table S2.** Comparison between the responsivities our PEC-type GaS photodetectors and other UV-sensitive photodetectors based on solution-processed 2D materials.

| **Materials** | **Device configuration** | **Measurement conditions** | | **Responsivity (mA W$^{-1}$)** | **Illumination intensity (mW cm$^{-2}$)** | **Wavelength (nm)** | **Ref.** |
|---|---|---|---|---|---|---|---|
| | | **Electrolyte** | **Applied potential (V *vs.* RHE) or voltage (V)** | | | | |
| GaS flakes | PEC-type | 1 M $H_2SO_4$ | - | 1.8 | 1.3 | 275 | This work |
| | | 1 M $Na_2SO_4$ | - | 4.6 | 1.3 | 275 | |
| | | 1 M KOH | - | 6.8 | 1.3 | 275 | |
| Graphene quantum dots | Metal/semiconductor/metal | - | 5 | 2.1 | 0.042 | 254 | [13] |
| Reduced-Graphene Oxide (RGO) | Metal/semiconductor/metal | - | 1 | 120 | 0.3 | 360 | [14] |
| ZnS-$MoS_2$ hybrid | Metal/semiconductor/metal | - | 1 | $9.4 \times 10^{-3}$ | 19.1 | 365 | [15] |
| 2D $MoS_2$-carbon quantum dot | Metal/semiconductor/metal | | 1 | 5.9 | ~1.9 | 365 | [16] |
| $WS_2$ | Metal/semiconductor/metal | | 25 | -0.08 (negative photoconductivity) | 200 | 365 | [17] |

| | | | | | | | |
|---|---|---|---|---|---|---|---|
| $WS_2$-RGO | Metal/semiconductor/metal | | 1.75 | 3.2 | 200 | 365 | 17 |

## 9. Stability of PEC-type GaS photodetectors

To evaluate the stability of GaS photodetectors, subsequent LSV scans were performed on the GaS photodetectors. As shown in **Figure S7a**, the GaS photoelectrodes show Raman spectra similar to the one measured for the as-produced GaS flakes, which means that the GaS flakes retain their starting structural properties during the PEC tests. **Figure S7b** reports the responsivities of the GaS photoelectrodes measured as a function of the number of the LSV scans. The devices exhibit the most stable PEC performance in 1 M KOH, showing a progressive responsivity stabilization over the first 10 LSV scans and a responsivity retention of 49.9% after 20 LSV scans. Since Raman analysis did not reveal any structural degradation of the GaS flakes, the causes of the possible degradation may be ascribed to the mechanical delamination induced by progressive gas evolution (*i.e.*, oxygen evolution due to OER), as previously reported for similar architectures based on other types of transition metal monochalcogenides (*e.g.*, GaSe[18] and GeSe[19]).

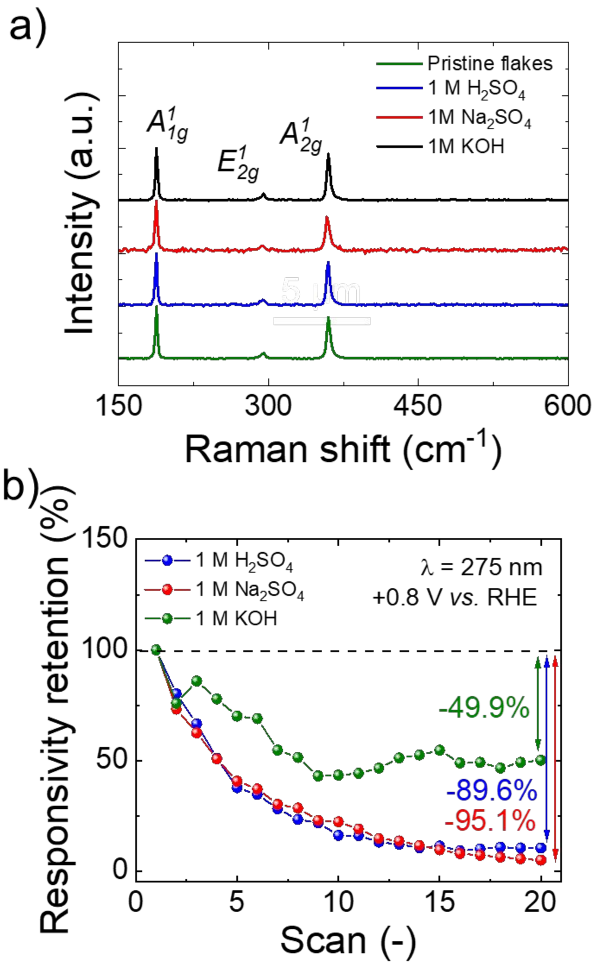


**Figure S7.** a) Raman spectra of the GaS photoelectrode after PEC stability tests (20 LSV scans) in the investigated electrolytes (1 M $H_2SO_4$, 1 M $Na_2SO_4$, and 1 M KOH), in comparison with the Raman spectrum of the pristine GaS flakes. b) Responsivity of the GaS photodetectors in the investigated electrolytes as a function of the number of the LSV scan.

## References


(1) Marvan, P.; Mazánek, V.; Sofer, Z. Shear-Force Exfoliation of Indium and Gallium Chalcogenides for Selective Gas Sensing Applications. *Nanoscale* **2019**, *11* (10), 4310–4317.

(2) Bonaccorso, F.; Bartolotta, A.; Coleman, J. N.; Backes, C. 2D-Crystal-Based Functional Inks. *Adv. Mater.* **2016**, *28* (29), 6136–6166.

(3) Ciesielski, A.; Samorì, P. Graphene via Sonication Assisted Liquid-Phase Exfoliation. *Chemical Society Reviews*. The Royal Society of Chemistry 2014, 381–398.

(4) Nicolosi, V.; Chhowalla, M.; Kanatzidis, M. G.; Strano, M. S.; Coleman, J. N. Liquid Exfoliation of Layered Materials. *Science (80-. ).* **2013**, *340* (6139).

(5) Khan, U.; O'Neill, A.; Porwal, H.; May, P.; Nawaz, K.; Coleman, J. N. Size Selection of Dispersed, Exfoliated Graphene Flakes by Controlled Centrifugation. *Carbon N. Y.* **2012**, *50* (2), 470–475.

(6) Backes, C.; Abdelkader, A. M.; Alonso, C.; Andrieux-Ledier, A.; Arenal, R.; Azpeitia, J.; Balakrishnan, N.; Banszerus, L.; Barjon, J.; Bartali, R.; et al. Production and Processing of Graphene and Related Materials. *2D Mater.* **2020**, *7* (2), 22001.

(7) Lu, Y.; Chen, J.; Chen, T.; Shu, Y.; Chang, R. J.; Sheng, Y.; Shautsova, V.; Mkhize, N.; Holdway, P.; Bhaskaran, H.; Warner, J. H. Controlling Defects in Continuous 2D GaS Films for High-Performance Wavelength-Tunable UV-Discriminating Photodetectors. *Adv. Mater.* **2020**.

(8) Jastrzebski, C.; Olkowska, K.; Jastrzebski, D. J.; Wierzbicki, M.; Gebicki, W.; Podsiadlo, S. Raman Scattering Studies on Very Thin Layers of Gallium Sulfide (GaS) as a Function of Sample Thickness and Temperature. *J. Phys. Condens. Matter* **2019**, *31* (7), 075303.

(9) Curreli, N.; Serri, M.; Spirito, D.; Lago, E.; Petroni, E.; Martín-García, B.; Politano, A.; Gürbulak, B.; Duman, S.; Krahne, R.; Pellegrini, V.; Bonaccorso, F. Liquid Phase Exfoliated Indium Selenide Based Highly Sensitive Photodetectors. *Adv. Funct. Mater.* **2020**, *30* (13), 1908427.

(10) Mukherjee, B.; Cai, Y.; Tan, H. R.; Feng, Y. P.; Tok, E. S.; Sow, C. H. NIR Schottky Photodetectors Based on Individual Single-Crystalline GeSe Nanosheet. *ACS Appl. Mater. Interfaces* **2013**, *5* (19), 9594–9604.

(11) Buscema, M.; Groenendijk, D. J.; Blanter, S. I.; Steele, G. A.; Van Der Zant, H. S. J.; Castellanos-Gomez, A. Fast and Broadband Photoresponse of Few-Layer Black Phosphorus Field-Effect Transistors. *Nano Lett.* **2014**, 14, 6, 3347–3352.

(12) Lopez-Sanchez, O.; Lembke, D.; Kayci, M.; Radenovic, A.; Kis, A. Ultrasensitive Photodetectors Based on Monolayer MoS 2. *Nat. Nanotechnol.* **2013**, 8, 497–501.

(13) Zhang, Q.; Jie, J.; Diao, S.; Shao, Z.; Zhang, Q.; Wang, L.; Deng, W.; Hu, W.; Xia, H.; Yuan, X.; Lee, S.-T. Solution-Processed Graphene Quantum Dot Deep-UV Photodetectors. *ACS Nano* **2015**, *9* (2), 1561–1570.

(14) Chitara, B.; Krupanidhi, S. B.; Rao, C. N. R. Solution Processed Reduced Graphene Oxide Ultraviolet Detector. *Appl. Phys. Lett.* **2011**, *99* (11), 113114.

(15) Gomathi, P. T.; Sahatiya, P.; Badhulika, S. Large-Area, Flexible Broadband Photodetector Based on ZnS-MoS 2 Hybrid on Paper Substrate. *Adv. Funct. Mater.* **2017**, *27* (31), 1701611.

(16) Sahatiya, P.; Jones, S. S.; Badhulika, S. 2D MoS2–Carbon Quantum Dot Hybrid Based Large Area, Flexible UV–Vis–NIR Photodetector on Paper Substrate. *Appl. Mater. Today* **2018**, *10*, 106–114.

(17) Shelke, N. T.; Karche, B. R. Hydrothermal Synthesis of WS2/RGO Sheet and Their Application in UV Photodetector. *J. Alloys Compd.* **2015**, *653*, 298–303.

(18) Zappia, M. I.; Bianca, G.; Bellani, S.; Serri, M.; Najafi, L.; Oropesa-Nuñez, R.; Martín-García, B.; Bouša, D.; Sedmidubský, D.; Pellegrini, V.; Sofer, Z.; Cupolillo, A.; Bonaccorso, F. Solution-Processed GaSe Nanoflake-Based Films for Photoelectrochemical Water Splitting and Photoelectrochemical-Type Photodetectors. *Adv. Funct. Mater.* **2020**, *30* (10), 1909572.

(19) Bianca, G.; Zappia, M. I.; Bellani, S.; Sofer, Z.; Serri, M.; Najafi, L.; Oropesa-Nuñez, R.; Martín-García, B.; Hartman, T.; Leoncino, L.; Sedmidubský, D.; Pellegrini, V.; Chiarello, G.; Bonaccorso, F. Liquid-Phase Exfoliated GeSe Nanoflakes for Photoelectrochemical-Type Photodetectors and Photoelectrochemical Water Splitting. *ACS Appl. Mater. Interfaces* **2020**, *12* (43), 48598–48613.
